# Phase Inversion and Interfacial Layer Microstructure in Emulsions Stabilized by Glycosurfactant Mixtures

**DOI:** 10.3390/nano11020331

**Published:** 2021-01-27

**Authors:** Rodolfo Esposito, Domenico Cavasso, Marcella Niccoli, Gerardino D’Errico

**Affiliations:** 1 Department of Chemical Sciences, University of Naples Federico II, Via Cintia 4, Complesso Universitario di Monte Sant’Angelo, I-80126 Naples, Italy; rodolfo.esposito@unina.it (R.E.); domenico.cavasso@unina.it (D.C.); marcella.niccoli@unina.it (M.N.); 2 Consorzio Interuniversitario per lo Sviluppo dei Sistemi a Grande Interfase (CSGI), Via della Lastruccia 3, I-50019 Florence, Italy

**Keywords:** emulsion, sugar-based surfactant, pseudo-phase diagram, electron paramagnetic resonance

## Abstract

Identification of strategies to prolong emulsion kinetic stability is a fundamental challenge for many scientists and technologists. We investigated the relationship between the emulsion stability and the surfactant supramolecular organization at the oil–water interface. The pseudo-phase diagrams of emulsions formed by water and, alternatively, a linear or a branched oil, stabilized by mixtures of two sugar-based surfactants, Span80 and Tween80, are presented. The surfactant ordering and dynamics were analyzed by electron paramagnetic resonance (EPR) spectroscopy. In Oil-in-Water (O/W) emulsions, which are stable for more than four days, disordered surfactant tails formed a compact and viscous layer. In Water-in-Oil (W/O) emulsions, whose stability is much lower, surfactants formed an ordered layer of extended tails pointing toward the continuous apolar medium. If linear oil was used, a narrow range of surfactant mixture composition existed, in which emulsions did not demix in the whole range of water/oil ratio, thus making it possible to study the phase inversion from O/W to W/O structures. While conductometry showed an abrupt inversion occurring at a well-defined water/oil ratio, the surfactant layer microstructure changed gradually between the two limiting situations. Overall, our results demonstrate the interconnection between the emulsion stability and the surfactant layer microstructuring, thus indicating directions for their rational design.

## 1. Introduction

Emulsions are kinetically stable dispersions of liquid droplets (oil or water) in another nonmiscible continuous liquid phase (water or oil). The oil–water interfacial tension is usually lowered by adding one or more emulsifying agents to the mixture. Although various alternatives are known (e.g., polymers, solid particles), surfactants remain by far the most used emulsifiers in industrial formulations [1,2,3]. Surfactants form a monolayer at the droplet surface, exposing their polar head groups to water, while the nonpolar tails point towards the oil phase [1].

When the droplets diameter is low (i.e., it ranges 50–200 nm) these microheterogeneous systems are termed nanoemulsions [4,5]. The small droplet size assures long kinetic stability, since Brownian motion overcomes the gravity forces that drive the system separation, avoiding creaming and sedimentation. The coexistence of hydrophobic, hydrophilic and amphiphilic domains enables emulsions, and specifically nanoemulsions, to solubilize a wide range of substances. Therefore, these systems have found numerous applications over a wide range of areas such as pharmaceuticals [6], foods [7], cosmetics [8], agrochemicals [9] and fuels [10], and new applications are continuously being reported [5,11].

In spite of the long standing scientific and technological interest in these systems, their physico-chemical characterization remains a fundamental challenge for many research groups. Emulsion and nanoemulsion macrostructure and stability critically depend on the molecular interactions and supramolecular organization of the surfactants at the oil–water interface [12]. In turn, these depend on the combination of a large number of factors, including temperature, oil type, oil and water relative amounts, surfactant(s) type and concentration.

Sorbitan esters (commercially known as Span surfactants) are among the most employed emulsifiers [13,14]. The properties of these surfactants can be finely tuned by changing the length and saturation of the acyl chain [15]. Moreover, their hydrophilicity can be increased by decorating the sugar ring with poly(ethylene glycol) chains (resulting in the Tween surfactant series). These sugar-based nonionic surfactants are recognized as safe and biocompatible products; they are affected neither by pH changes of the mixture nor by the presence of other components [16,17].

Surfactant mixtures are more effective for emulsion stabilization with respect to single surfactants [7,18] since their composition can be finely tuned depending on conditions and requirements. Mixtures of Span80 and Tween80, two terms of the abovementioned surfactant series, have been used to formulate both Oil-in-Water (O/W) and Water-in-Oil (W/O) emulsions [15,19]. Specifically, mixtures of these surfactants have been recently demonstrated to effectively stabilize nanoemulsions [20,21,22,23,24]. The macrostructure of these systems has been characterized in terms of droplet size and distribution [24,25]; interestingly, it has been found that oils with branched chain favor the formation of nanoemulsions with small-size droplets [26]. The kinetic stability has also been investigated. On the other hand, the surfactant organization at the droplet interface has received less attention.

In this work, we report and analyze the pseudo-phase diagrams of emulsions formed by water and, alternatively, a linear or a branched oil stabilized by mixtures of Span80 and Tween80. Firstly, we determined the pseudo-phase diagrams, delimiting composition regions in which samples showed a uniform milky appearance with no evidence of demixing, as a function of time.

Moreover, we investigated the surfactant microscopic organization and dynamics by electron paramagnetic resonance (EPR) spectroscopy. EPR is a powerful tool in physico-chemical characterization of supramolecular surfactant aggregates since it is sensitive and nondestructive; furthermore, it requires only a small sample size. By using purposely tailored molecular probes, the EPR technique has proved to provide detailed information on the structure and dynamics of micelles [27], polymer–surfactant aggregates [28] and vesicles [29]. It has also been used to analyze the properties of surfactants layers adsorbed at the solid/liquid and liquid/liquid interfaces, as in emulsions, microemulsions [30,31,32,33,34,35], nanoemulsions [7] and reversed micelles [36,37]. Specifically, in the present study we characterize by EPR the Span80/Tween80 layer interposing between water and oil domains by using two amphiphilic spin-labeled fatty acids, 5- and 16-doxyl stearic acids (abbreviated as 5- and 16-DSA, respectively). Spectral features of these probes provided information on the microenvironment polarity, as well as on the surfactant orientation, mobility and segmental ordering of the acyl chains.

Our efforts aimed to get a better understanding of the relationship between the surfactant microscopic organization at the droplet boundary, the system structure at the nanoscopic scale and the emulsion stability over time, thus contributing to build a robust scientific platform for a rational design of emulsion formulations.

## 2. Materials and Methods

### 2.1. Materials

**Figure 1:**
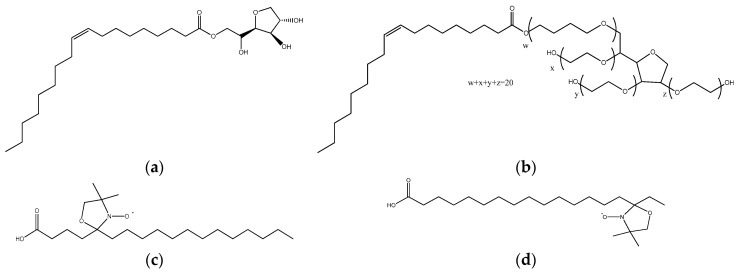
Molecular formulae of (**a**) Span80, (**b**) Tween80, (**c**) 5-doxyl stearic acid (5-DSA) and (**d**) 16-doxyl stearic acid (16-DSA).

The molecular formulae of some substances used in this study are presented in Figure 1. Surfactants Span80 and Tween80 were purchased from Sigma-Aldrich (St. Louis, MO, USA). Span and Tween are registered trademarks of Croda Inc. (New Castle, DE, USA).

Span80 (sorbitan monooleate) is a biodegradable surfactant obtained from the natural fatty acid oleic acid and the sugar alcohol sorbitol; it is a viscous, brown, liquid, lipophilic surfactant with low hydrophilic–lipophilic balance (*HLB_Span80_* = 4.3). Tween80 (polyethylene glycol sorbitan monooleate) is a hydrophilic derivative of Span80, obtained from polyethoxylated sorbitan and oleic acid, with an amber, sticky liquid appearance and *HLB_Tween80_* = 15.

Two oils were tested for emulsion preparation: one mainly composed by linear alkanes and another one containing a large fraction of branched alkanes. Below, they are named L.O. (linear oil) and B.O. (branched oil), respectively. Both oils present a negligible content of aromatics and unsaturated chains and are polydisperse samples with an average number of carbon atoms per molecule equal to ~12. The former one, commercially called Alboles 2701 (from Lubra S.p.A., Cornaredo, MI, Italy), is a mixture of linear aliphatic hydrocarbons with a distillation range between 180 and 240 °C. Its kinematic viscosity at 40 °C is 1.8 mm^2^ s^−1^ and the density at 15 °C is 0.78 kg dm^−3^. The latter one, commercially called Isopar G (from Exxon Mobil Chemicals, Parkway Spring, TX, USA), is mainly composed of isoalkanes with a distillation range comprised between 165 and 177 °C. The kinematic viscosity at 40 °C is 1.19 mm^2^ s^−1^ and the density at 15 °C is 0.75 kg dm^−3^.

The spin-labeled fatty acids 5-doxyl stearic acid (5-(1-oxyl-2,2-dimethyl-oxazolidin) stearic acid, 5-DSA) and 16-doxyl stearic acid (16-(1-oxyl-2,2-dimethyloxazolidin) stearic acid, 16-DSA), used for EPR measurements, were also purchased from Sigma-Aldrich and stored at −20 °C. Products were used without further purification. All aqueous solutions were prepared by using Millipore water.

### 2.2. Samples Preparation

The preparation of emulsion samples consisted of the following steps:

1. Proper amounts of oil (either L.O. or B.O.) and Span80 were weighted in a vial and thoroughly mixed by using a bench-type vortex (ArgoLab Mix, Carpi, Italy).
2. Proper amounts of water and Tween80 were weighted in another vial and thoroughly mixed by vortexing. The pH of this solution was checked to be from neutral to weakly acid (pH = 6–7).
3. The contents of the two vials were combined, briefly vortexed and then sonicated using a Sonics Vibracell VCX 130 PB ultrasonic processor (Sonics&Materials, Newtown, CT, USA) equipped with a 3 mm titanium probe running at an amplitude of 40% for 20 min in an ice bath.

### 2.3. Pseudo-Phase Diagram Determination

Two systems were investigated: water/L.O./(Tween80+Span80) and water/B.O./(Tween80 + Span80). The relative amount of the two solvents present in each sample (water and oil) was quantified as the oil weight percent with respect to the total solvent (water and oil), denoted by *L.O.%* and *B.O.%*, respectively.

In all the samples the total amount of the surfactant mixture (Tween80+Span80) was fixed to 4% in weight with respect to the total sample amount. The relatively high total surfactant content chosen for the study should have minimized the effect of *L.O.%* and *B.O.%* variations on droplet dimensions [38,39]. Instead, the ratio between the two surfactants was varied. This variable was quantified as the Span80 fraction with respect to the total surfactant weight, indicated with *α_Span80_*. *α_Span80_* equal to 0.0 means that only Tween80 is present, while 1.0 stands for the presence of only Span80. For both the investigated systems, the entire *α_Span80_* range was analyzed.

Since emulsions are thermodynamically unstable and can evolve with time, all the samples (stored at 25 ± 1 °C) were monitored by the ocular inspection after 3, 24 and 96 h from preparation.

### 2.4. Conductometric Measurements

Systematic measurements of specific conductivity were performed on water/L.O./(Tween80 + Span80) samples using a CDM 210 conductometer (Radiometer Analytical, Villeurbanne, France) with a conductivity cell having a constant of 0.1 cm^−1^. Before measuring the conductivity of a sample, the cell was calibrated by determining the cell constant *K*. The cell constant was determined by measuring the conductivity of a KCl standard solution with a specific conductivity known with great accuracy at several concentrations and temperatures. The specific conductivity, *χ* (μS cm^−1^), was then obtained as the product of the cell constant and the conductivity of the solution. The conductometric measurements were performed in a thermostated room (25 ± 1 °C) using samples whose temperature was preconditioned by a thermo-cryostat at the same temperature.

### 2.5. EPR Characterization

EPR measurements were performed to characterize the surfactant layer in terms of local polarity, tail order and mobility by using two spin-labelled saturated fatty acids as probes: 5-DSA and 16-DSA. The former presents the cyclic nitroxide reporter group relatively close to surfactant polar head, while in the latter the label is at the acyl chain terminus. EPR spectral parameters of these probes are pH-dependent [31,36]. At the pH of the aqueous pools of the investigated emulsion, which was below the pKa of stearic acid (pKa = 7.45), the carboxylic group was in the un-dissociated form. To incorporate the probes in the samples, a proper emulsion amount, right after the sonication step, was added to a thin probe film to reach a probe concentration of 2% by mass with respect to the surfactants, shaken for 15 min and finally left at rest for other 15 min. The probe thin film had been previously obtained by drying under nitrogen the proper amount of a 1 mg mL^−1^ probe solution in ethanol placed in a round-bottom glass vial. The same procedure was repeated for the two spin probes.

Oxygen-induced line broadening in EPR spectra can occur when using apolar media such as oils [34]. For this reason, the probe-containing nanoemulsions were purged with nitrogen (10 min) before being transferred to a 25 μL glass capillary and flame sealed. The spectra were recorded within 3 h from sample preparation.

EPR spectroscopy of the labelled emulsions was performed using an X-band (9 GHz) Bruker Elexsys E-500 spectrometer (Rheinstetten, Germany). Capillaries containing the samples were placed in a standard 4 mm quartz sample tube to record measurements at room temperature (25 ± 1 °C). Spectra were recorded using the following instrumental settings: frequency 9.87 GHz; non-saturating incident power, 6.40 mW; sweep width, 140 G; center magnetic field 3510 G; resolution, 1024 points; time constant, 10.24 ms; conversion time, 20.48 ms; modulation frequency, 100 kHz; modulation amplitude, 1.0 G. Several scans, typically 128, were accumulated to improve the signal-to-noise ratio. The error in the determination of the distance between the peaks in ESR spectra depended on their width; the highest possible error was ±0.3%.

### 2.6. Statistical Analysis

Specific conductivity and EPR measurements were repeated at least thrice on independently prepared samples with the same nominal composition. The results are reported as mean value ± standard deviation.

## 3. Results and Discussion

### 3.1. Pseudo-Phase Diagrams

**Figure 2:**
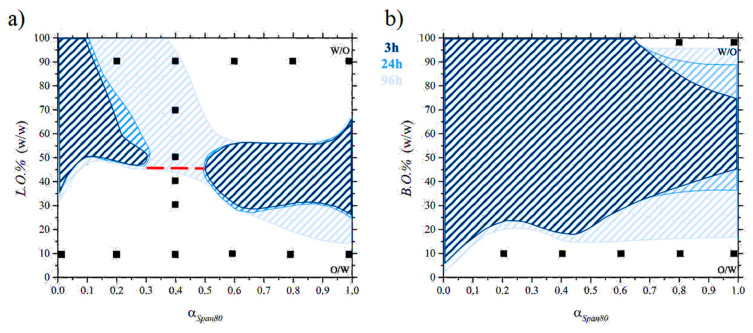
Pseudo-phase diagrams of the systems: (**a**) water/L.O. (linear oil)/(Tween80 + Span80) and (**b**) water/B.O. (branched oil)/(Tween80 + Span80) at 25 °C. The total surfactant amount was fixed to 4% in weight with respect to the total sample amount. Shadowed areas represent composition domains in which samples show macroscopic demixing when inspected after 3, 24 and 96 h from preparation, according to the reported legend. Black markers indicate the compositions of the samples analyzed by electron paramagnetic resonance (EPR). The red dashed line indicates the composition at which the inversion from O/W to W/O emulsions occurs, as detected by specific conductivity measurements.

The pseudo-phase diagrams of the systems water/L.O./(Tween80 + Span80) and water/B.O./(Tween80 + Span80) are shown in Figure 2a,b, respectively. The total amount of the surfactant mixture (Tween80 + Span80) is constant and equal to 4% in weight with respect to the total sample amount. The horizontal axes report *α_Span80_*, the weight fraction of Span80 with respect to the total surfactant amount. With increasing *α_Span80_*, the HLB of the surfactant mixture decreases from 15 (*α_Span80_* = 0.0) to 4.3 (*α_Span80_* is 1.0). The vertical axes report the oil weight percent on the total solvent (water + oil) weight (*L.O.%* and *B.O.%*, respectively). Composition regions in which the sample shows an evident macroscopic demixing are differently colored, depending on the time (3, 24 or 96 h from sample preparation) at which demixing was observed.

For the water/L.O./(Tween80 + Span80) system, two different regions of instability were detected after 3 h (shaded in deep blue in Figure 2a). The first one, on the left-hand-side of the diagram (*α_Span80_* < 0.3), extends from *L.O.%* equal to 35 up to 100. This indicates that surfactant mixtures with a Tween80 prevalence are not able to stabilize oil-rich emulsions. The second region of instability is on the right-hand-side of the diagram (*α_Span80_* > 0.5), and it extends from *L.O.%* = 25 up to 65. Thus, surfactant mixtures in which Span80 prevails are able to stabilize emulsions rich in one of the two solvents, while samples in which the oil and water amounts are similar rapidly demix. To the exclusion of these regions, the pseudo-phase diagram shows a large double-T shaped stability area, in which the samples present a uniformly milky aspect (see Figure S1). Water-rich emulsions (at the bottom of the figure) are stable in the entire *α_Span80_* and presumably present a O/W structure. Oil-rich emulsions, with a putative W/O structure, are stable above *α_Span80_* = 0.1. Samples with intermediate *L.O.%* show a higher tendency to demix.

**Figure 3:**
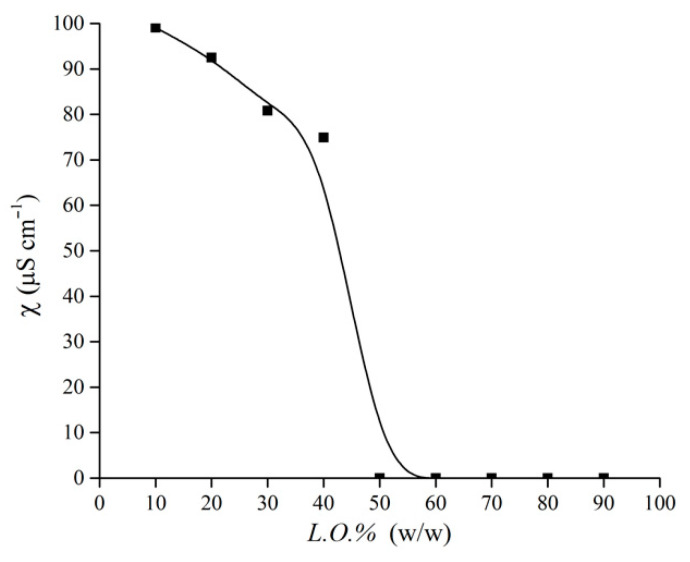
Specific conductivity of water/L.O./(Tween80 + Span80) emulsions as a function of the oil content *L.O.%* at 25 °C. The total surfactant amount was fixed to 4% in weight with respect to the total sample amount, and surfactant composition was *α_Span80_* = 0.4 in all samples.

Interestingly, for 0.3 < *α_Span80_* < 0.5 (11.8 < HLB < 9.7), the emulsions are stable at all the *L.O.%* values. To investigate how the emulsion structure changes with increasing *L.O.%*, we performed a conductometric investigations on samples at *α_Span80_* = 0.4. The specific conductivity trend, shown in Figure 3, presents an abrupt decrease above *L.O.%* = 40. Indeed, at higher oil contents almost null values are detected. These results clearly highlight a sudden inversion of the emulsion from a O/W to a W/O structure, with no evidence of an intermediate bicontinuous organization. In the pseudo-phase diagram, the oil content at which the inversion occurs is indicated by a red dashed line.

After one day from the sample preparations, only a modest enlargement of the instability regions is observed (see the middle blue areas in Figure 2a).

Four days are sufficient to observe a significant widening of the instability regions, which merge into a single zone (shaded in light blue), separating stability regions of O/W and W/O emulsions. At the bottom of the figure, at low *α_Span80_*, the O/W stability is guaranteed for *L.O.%* up to around 45 (see Figure S2). As the *α_Span80_* increases, the *L.O.%* at which demixing is observed decreases to about 15. At the top of the figure, the W/O stability area shows that surfactant mixtures with *α_Span80_* >0.5 are able to stabilize W/O emulsions in which the water content is about 40 wt%.

In the pseudo-phase diagram of the system water/B.O./(Tween80 + Span80), shown in Figure 2b, a large instability region is already observed after 3 h from sample preparation. Two domains in which the samples do not demix are present, occupying the bottom and the top of the diagram, respectively. In the lower part of the diagram, O/W emulsions are stable in the entire *α_Span80_* investigated range. The amount of B.O. that can be included in this system increases from 5% to 45% with increasing the Span80 surfactant fraction. In the higher part of the diagram, W/O emulsions are stable only for *α_Span80_* ≥ 0.5. The amount of water increases, reaching 25% in samples where only Span80 is present as surfactant.

After 24 h, the instability zone tends to expand on the right-hand-side side; see the middle blue zone in Figure 2b. A four-day aging leads to a notable widening of the instability region, shaded in light blue. Only a very narrow range of W/O emulsion stability remains for *B.O.%* > 95. In the bottom of the O/W, structures are stable for *B.O.%* < 15 at *α_Span80_* > 10%.

### 3.2. EPR Results

The structure and dynamics of the interfacial surfactant layer in water/L.O./(Tween80 + Span80) and water/B.O./(Tween80 + Span80) emulsions were investigated by EPR spectroscopy. Compositions of the considered samples are marked with black squares in Figure 2a,b. Two spin labelled fatty acids were employed as probes: 5-DSA and 16-DSA [7]. Being amphiphilic, these probes are embedded into the surfactant layer, and their spectroscopic features furnish detailed information on the microenvironment in which the paramagnetic label resides. 5-DSA monitors the surfactant structuring and dynamics at the oil–water interface. In 16-DSA, the label position allows the exposure of the surfactant tail to the oil phase to be investigated.

**Figure 4:**
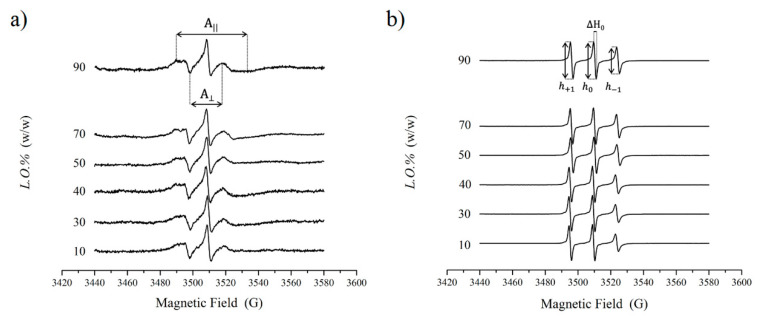
Electron paramagnetic resonance (EPR) spectra of 5-doxyl stearic acid (5-DSA) (**a**) and 16-doxyl stearic acid (16-DSA) (**b**) in water/L.O./(Tween80 + Span80) emulsions at 25 °C, recorded within 3 h from sample preparation. The total surfactant amount was fixed to 4% in weight with respect to the total sample amount and surfactant compositions was *α_Span80_* = 0.4 in all samples. The oil content was progressively increased as indicated by the labels in the figures.

Figure 4a,b shows the EPR spectra of 5-DSA and 16-DSA in water/L.O./(Tween80 + Span80) emulsions at constant surfactant mixture composition (*α_Span80_* = 0.4) at an increasing relative amount of the oil phase (10 ≤ *L.O.%* ≤ 90). The spectra are constituted by the three hyperfine lines due to the coupling between the unpaired electron spin, with spin equal to 1/2 and the nitrogen ^14^N, with nuclear spin equal to 1. 16-DSA spectra are characterized by the presence of three lines of unequal heights and widths; in the case of 5-DSA, the low- and high-field lines are further broadened and partially split in two components. These results demonstrate that the spin probes are embedded in the surfactant layer at the oil–water interface and preferentially rotate around the long molecular axis [40]. For both probes, no evidence of superimposed spectra arising for the probe partitioning between different environments is observed, thus suggesting Span80 and Tween80 to be randomly distributed at the interface, with no formation of domains with different local surfactant composition. The lineshape differences arise from the different rotation rates: in the case of 16-DSA, the anisotropic motion of the nitroxide moiety is sufficiently rapid to average the hyperfine interaction, while for 5-DSA the motion is much slower [37]. Thus, the label mobility within the surfactant monolayer is significantly more hindered close to the surfactant heads. Inspection of the figures shows, for both spin probes, no dramatic change with increasing the oil content in the system, indicating that the interfacial surfactant layer is only slightly perturbed; particularly, it does not undergo evident structural reorganization upon emulsion phase inversion.

Quantitative analysis of 5-DSA spectra allows the evaluation of the hyperfine coupling constant, $A_{N}$, and the order parameter, *S*. $A_{N}$ is an index of the micropolarity experienced by the nitroxide label. *S*, which measures the label wobbling mobility, depends on the spatial ordering of the labeled segment of the probe tail with respect to the normal to the surfactant monolayer surface. These parameters can be evaluated from the distance (in G) between maxima and minima of the spectra, (see Figure 4a) according to [34]:

$$A_{N}=\frac{1}{3}\left(A_{∥}+2A_{⏊}\right)$$

$$S=\frac{A_{∥}-A_{⏊}}{A_{\mathrm{zz}}-\frac{1}{2}(A_{\mathrm{xx}}+A_{\mathrm{yy}})}\frac{A_{N}^{0}}{A_{N}}$$

$A_{\mathrm{xx}}$, $A_{\mathrm{yy}}$ and $A_{\mathrm{zz}}$ are the principal elements of the real hyperfine splitting tensor in the spin Hamiltonian of the spin-label, and $A_{N}^{0}$ is the isotropic hyperfine coupling constant in crystal state. The values of these parameters are reported in the literature [41].

**Figure 5:**
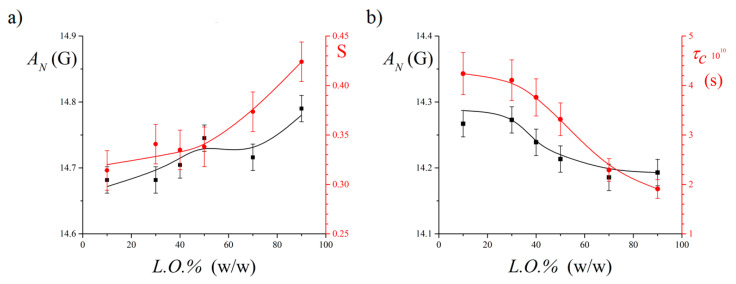
Dependence on oil content *L.O.%* of the EPR spectroscopic parameters of 5-DSA (**a**) and 16-DSA (**b**) in water/L.O./(Tween80 + Span80) emulsions at 25 °C. For both spin-probes, the hyperfine coupling constant *A_N_* is reported (on the left-hand side ordinate). The right-hand side ordinate reports the order parameter, *S*, and the correlation time, $\tau_{c}$, for 5-DSA and 16-DSA, respectively. The total surfactant amount was fixed to 4% in weight with respect to the total sample amount and surfactant compositions was *α_Span80_* = 0.4 in all samples.

Overall, the $A_{N}$ and *S* values do not show dramatic change with increasing the oil content in the system (Figure 5a). The *S* value is typical of 5-DSA inserted in quite ordered surfactant layers [7,31,42] and weakly increases with *L.O.%*, indicating an increase of the order of the surfactant tails close to the headgroups. These data confirm that no bicontinuous mesophase forms in the investigated system: in cases of phase inversion occurring through the intermediate formation of a bicontinuous structure, the order parameter passes through a maximum [31], which is not observed here. Surprisingly, even the $A_{N}$ value slightly increases, indicating that the nitroxide label experiences a more polar environment. This suggests that it becomes progressively more exposed to the aqueous medium.

Regarding 16-DSA, whose spectra consist of three well-defined lines, the $A_{N}$ values can be directly derived as the distance among them. In addition, the tumbling correlation time of the spin-probe, $\tau_{c}$, can be derived, which reflects the label rotational mobility, as determined by the microenvironment viscosity. $\tau_{c}$ is obtainable by the relation [35]:

$$\tau_{c}=6\times 10^{-10}\Delta H_{0}\left[{\left(\frac{h_{0}}{h_{+1}}\right)}^{1/2}+\;{\left(\frac{h_{0}}{h_{-1}}\right)}^{1/2}-2\right]$$

$h_{+1}$, $h_{0}$ and $h_{-1}$ are the intensities of the low-field, central- and high-field lines of the EPR spectrum, respectively, and $\Delta H_{0}$ is the width of the central line (see Figure 4b). This equation is applicable for rotational correlation times in the range 10^−11^ < $\tau_{c}$ < 3 × 10^−9^ s.

The $\tau_{c}$ values decrease (Figure 5b), indicating that the rotational mobility of the terminal part of the tails, to which the nitroxide probe is linked, increases. This result suggests that the tail termini are in a less viscous environment.

Analysis of order, polarity and rotational parameters derived from EPR spectra suggests that, upon the interface curvature inversion due to the emulsion transition from an O/W to a W/O structure, the surfactant layer undergoes a progressive structural rearrangement. Both in O/W and in W/O emulsions, the polar heads position themselves at the interface. In O/W systems, the lipophilic chains converge toward the oil droplet interior, experiencing steric repulsion and entangling with each other. This results in a relatively disordered (see the 5-DSA *S* value) and blocked (see the 16-DSA $\tau_{c}$ value) surfactant molecule meso-structuring. Thus, surfactants form a compact layer, as also highlighted by the 5-DSA $A_{N}$ value, indicating the quite apolar environments experienced by the first segments of the acyl chains. This description of the surfactant layer is consistent with literature evidence reporting that amphiphiles assume bent and, more generally, disordered conformations in O/W aggregates [33]. In W/O systems, the chains radially point toward the external medium, experiencing less steric constraint. This leads to a more ordered and, at the same time, more dynamic surfactant organization, which also reflects in an enhanced water penetration among the surfactant headgroups and closer tail segments. This behavior can be figuratively described in terms of the “hedgehog” model [35]: by curving its back, the hedgehog can modify the relative position of its spines, increasing or decreasing the space between the edges of the spines.

While the increase of the *L.O.%* in the system causes significant changes of the EPR spectral parameters connected to phase inversion from a O/W to a W/O structure (see Figure 5), *α_Span80_* variations do not result in appreciable spectral changes (spectra not shown). This is an interesting result, which highlights that, in emulsions stabilized by Tween80 + Span80 mixtures, the microstructure of the hydrophobic domain formed by the surfactant tails is poorly affected by changes of the headgroup layer composition, provided that the surface curvature, mainly determined by the solvent ratio, remains unaltered. Average values of $A_{N}$, S and $\tau_{c}$, determined in water/L.O./(Tween80+Span80) emulsions with *L.O.%* equal to 10 (O/W) and 90 (W/O) and *α_Span80_* varying in the range in which samples present a uniform appearance, are collected in Table 1.

Inspection of the table shows that the comments done at *α_Span80_* = 0.4 concerning the two different emulsions structures (O/W and W/O) can be extended to the other *α_Span80_* investigated. Indeed, the difference between the $A_{N}$ values observed for 5-DSA and 16-DSA is much lower in O/W emulsions than in W/O ones. This means that the acyl chains assume a less extended conformation in the former systems than in the latter ones. A further confirmation comes from the lower *S* value determined for 5-DSA, indicating a less ordered assembly of the tails. Moreover, the higher $\tau_{c}$ of 16-DSA suggests that the label has a more hindered motion.

In Table 1, the $A_{N}$, S and $\tau_{c}$ determined in water/B.O./(Tween80 + Span80) emulsions are also reported. Even in this system the oil content was found to be the main parameter affecting the spin probes spectral parameters. The $A_{N}$ values observed in the water/L.O./(Tween80 + Span80) and water/B.O./(Tween80 + Span80) samples with the same oil content are almost the same. In contrast, the *S* values determined for 5-DSA are significantly lower for the systems in which the branched oil was used. Moreover, it is to be highlighted that *S* is higher in O/W emulsions than in W/O ones, a variation opposite to that observed when the linear oil was used.

This interesting evidence could be ascribed to the bulkiness of the branched oil, whose insertion among the surfactant tails is, at least partially, hindered. Consequently, the surfactant tails are less able to point in the extended conformation toward the oil protruding in it. Instead, they are forced to assume twisted conformations, leading to a less-ordered surfactant layer. Even the $\tau_{c}$ values obtained for 16-DSA are lower for the systems in which the branched oil was used. A possible reason for this last evidence could be the lower viscosity of the branched oil with respect to the linear one. Here, it is important to highlight that both the lower compactness of the surfactant tails and the increased droplet mobility deriving from the lower viscosity are likely to play an important role in destabilizing the water/B.O./(Tween80 + Span80) emulsions (particularly the W/O ones), whose stability domains are much narrower with respect to those of the water/L.O./(Tween80 + Span80) ones (see Figure 2).

## 4. Conclusions

Emulsions are complex systems whose macroscopic stability is strictly related to the microscopic structure of the surfactant layer adsorbed at interface between the dispersed droplets and the continuous medium [43].

In this work, we studied the pseudo-phase diagrams of emulsions formed by water and, alternatively, a linear or a branched mineral oil stabilized by mixtures of Span80 and Tween80. Moreover, we investigated the surfactant microscopic organization at the droplet interface by EPR spectroscopy.

The pseudo-phase diagrams show that Span80+Tween80 mixtures are able to stabilize O/W emulsions in whole range of surfactant mixture composition, quantified as *α_Span80_*. Accordingly, the EPR results show that the microstructure of the surfactant layer adsorbed at the interface between oil and water is almost unaffected by the change in mass ratio between the two glycosylated surfactants. Span80 and Tween80 present the same hydrophobic tail, while the hydrophilic head of the latter one is considerably bulkier. The surface of O/W droplets, which present a positive curvature, are able to accommodate both type of headgroups with negligible effects of the surfactant tail arrangement.

W/O emulsions appear less stable than O/W ones; particularly, only surfactant mixtures enriched in Span80 show emulsifying ability. In this case, the negative curvature of the droplets reduces the space available to the headgroups, and consequently, surfactants with smaller head are favored. In the *α_Span80_* range in which emulsion are stable, surfactant mixture composition scarcely affects the microstructure of the hydrophobic layer formed by the surfactant tail.

Emulsions containing similar amounts of oil and water generally show a limited stability. Nonetheless, when the linear oil is used, samples are stable in the intermediate *α_Span80_* range, going from *α_Span80_* = 0.3 to 0.5.

Overall, these results confirm that glycosurfactants are a good emulsifier, flexibly able to adapt to different types of mesostructures. Particularly, if rationally used, they are able to also stabilize W/O emulsions, whose industrial and commercial interest is continuously growing. Thus, they are effective green alternatives to conventional surfactants currently in use in formulative practice.

The microstructure of the surfactant layer is mainly determined by the mass ratio between the two solvents, quantified as *L.O.%* and *B.O.%*. In the emulsions formulated with the linear oil, the change of microstructure can be followed by progressively increasing *L.O.%* in samples with *α_Span80_* = 0.4. In O/W emulsions, the tails of the surfactants adsorbed at the droplet interface form a disordered layer in which the mobility of the entangled chains is hindered. In W/O emulsions, the tails form an ordered array of extended chains freely pointing toward the external continuous apolar medium. The linear oil molecules can penetrate the surfactant layer, intercalating among the tails, leading to a strongly hydrophobic environment that is poorly permeable to water molecules. Interestingly, while the phase inversion form O/W to W/O structures abruptly occurs above *L.O.%* = 40 (as demonstrated by specific conductivity measurements), the surfactant layer microstructure shows a gradual variation, which can be pictorially figured according to the “hedgehog” model [35], in which the relative position of the spines is determined by the skin curvature. Thus, a small rearrangement of the surfactant layer microstructure corresponds to dramatic changes of the system nanostructure.

When branched oil is used for emulsion formulation, the overall stability decreases. Particularly, W/O emulsions demix in the whole composition range explored, with the exception of a small domain of the phase diagram at high *B.O.%* and *α_Span80_* values. Branched oils have been recently reported to lead to favor formation of emulsion with small-size droplets, which, however, are relatively unstable [26]. The high Ostwald ripening rate is likely to play a role in this instability [44], along with the low viscosity, which favors droplet impact (not hindered by any electrostatic interaction) [26]. Our results suggest a further reason for emulsion instability to be derived from the inability of the bulky branched oil to intercalate among the surfactant tail termini, hindering their extension toward the oil phase [45]. This results in an increased disorder and dynamics of the microscopic surfactant self-organization, possibly leading, on the macroscopic scale length, to a reduced droplet stability. On this basis, one could expect surfactants able to maintain a higher order, such as those bearing a saturated tail, to be better suited as emulsifiers of branched oils in water. Alternatively, a reduction of the tail dynamics could be obtained by using di- or branched-tailed surfactants, which should lead to a local crowding.

Our results show a clear relation between the surfactant layer microstructure and the emulsion structure at the nanoscopic scale, finally determining the system’s macroscopic stability. Specifically, surfactant tail ordering and dynamics are key parameters that have to be finely tuned on a rational basis in order to achieve emulsions designed to accomplish the requirements in the various application fields.

## Figures and Tables

**Table 1:** EPR spectral parameters of 5-DSA and 16-DSA in water/L.O./(Tween80 + Span80) and water/B.O./(Tween80 + Span80) emulsions at 25 °C.

System	Spin-Probe	$A_{N}$ (G)	*S*	$\tau_{c}\;10^{10}$ (s)
**water/L.O./(Tween80 + Span80)**				
*L.O.%* = 10 *α_Span80_* = 0.0–1.0 (O/W)	5-DSA	14.7 ± 0.2	0.35 ± 0.02	
	16-DSA	14.29 ± 0.05		4.5 ± 0.4
*L.O.%* = 90 *α_Span80_* = 0.2–1.0 (W/O)	5-DSA	14.8 ± 0.2	0.45 ± 0.03	
	16-DSA	14.13 ± 0.03		2.3 ± 0.6
**water/B.O./(Tween80 + Span80)**				
*B.O.%* = 10 *α_Span80_* = 0.2–1.0 (O/W)	5-DSA	14.6 ± 0.2	0.16 ± 0.04	
	16-DSA	14.3 ± 0.1		2.7 ± 0.6
*B.O.%* = 97 *α_Span80_* = 0.8–1.0 (W/O)	5-DSA	15.0 ± 0.1	0.07 ± 0.02	
	16-DSA	14.20 ± 0.02		0.7 ± 0.1

## Data Availability

The data presented in this study are available in the article or as supplementary materials.

## Supplementary Materials

The following are available online at https://www.mdpi.com/2079-4991/11/2/331/s1, Figure S1: Water/L.O./(Tween80+Span80) emulsion samples after 3 h from preparation. The total surfactant amount was fixed to 4% in weight with respect to the total sample amount and surfactant compositions was *α_Span80_* = 0.4 in all samples. The oil content *L.O.%* is indicated in the labels. Figure S2: Water/L.O./(Tween80+Span80) emulsion samples. The total surfactant amount was fixed to 4% in weight with respect to the total sample amount, surfactant composition and the oil content were *α_Span80_* = 0.4 and *L.O.%* = 40 in all the samples. Time from preparation is indicated in the labels. Figure S3: Electron paramagnetic resonance (EPR) spectra of 5-DSA (**a**) and 16-DSA (**b**) in water/B.O./ (Tween80+Span80) emulsions at 25 °C, recorded within 3 h from sample preparation. The total surfactant amount was fixed to 4% in weight with respect to the total sample amount and surfactant compositions was *α_Span80_* = 0.4 in all samples. The oil content is indicated in the labels of the figures.
