# Supplementary material for: Phase Inversion and Interfacial Layer Microstructure in Emulsions Stabilized by Glycosurfactant Mixtures
Source: Nanomaterials (Basel). 2021 Jan 27;11(2):331. doi: 10.3390/nano11020331 (PMC7911527; doi:10.3390/nano11020331)
Supplement: Supplementary file 1 [file nanomaterials-11-00331-s001.pdf]

Supplementary Material

Phase Inversion and Interfacial Layer Microstructure in Emulsions Stabilized by Glycosurfactant Mixtures

Rodolfo Esposito [1], Domenico Cavasso [1], Marcella Niccoli [1] and Gerardino D'Errico [1,2,*]

[1] Department of Chemical Sciences, University of Naples Federico II, Via Cintia 4, Complesso Universitario di Monte Sant'Angelo, I-80126 Naples, Italy; rodolfo.esposito@unina.it (R.E.); domenico.cavasso@unina.it (D.C.); marcella.niccoli@unina.it (M.N.)
[2] CSGI, Consorzio Interuniversitario per lo Sviluppo dei Sistemi a Grande Interfase, Via della Lastruccia 3, I-50019 Sesto Fiorentino, Florence, Italy
* Correspondence: gerardino.derrico@unina.it; Tel.: +39-081-674248

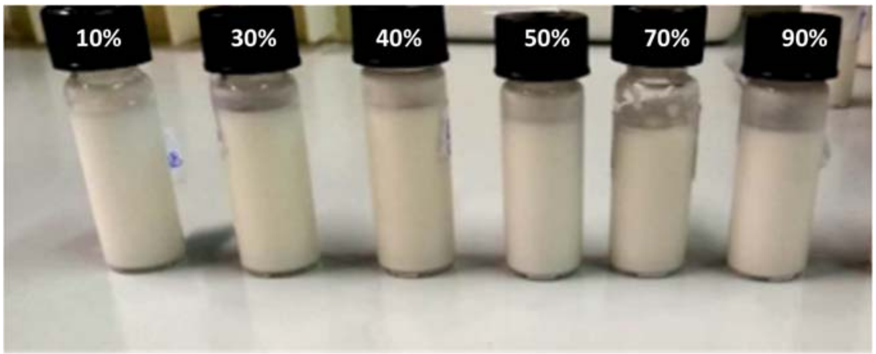

Figure S1. Water/L.O./(Tween80 + Span80) emulsion samples after 3 h from preparation. The total surfactant amount was fixed to 4% in weight with respect to the total sample amount and surfactant compositions was α_{Span80} = 0.4 in all samples. The oil content *L.O.%* is indicated in the labels.

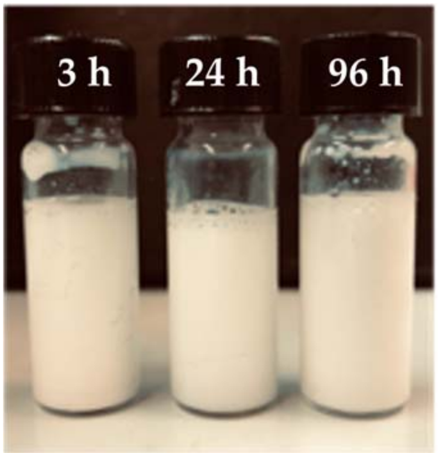

Figure S2. Water/L.O./(Tween80 + Span80) emulsion samples. The total surfactant amount was fixed to 4% in weight with respect to the total sample amount, surfactant composition and the oil content were α_{Span80} = 0.4 and *L.O.%* = 40 in all the samples. Time from preparation is indicated in the labels.

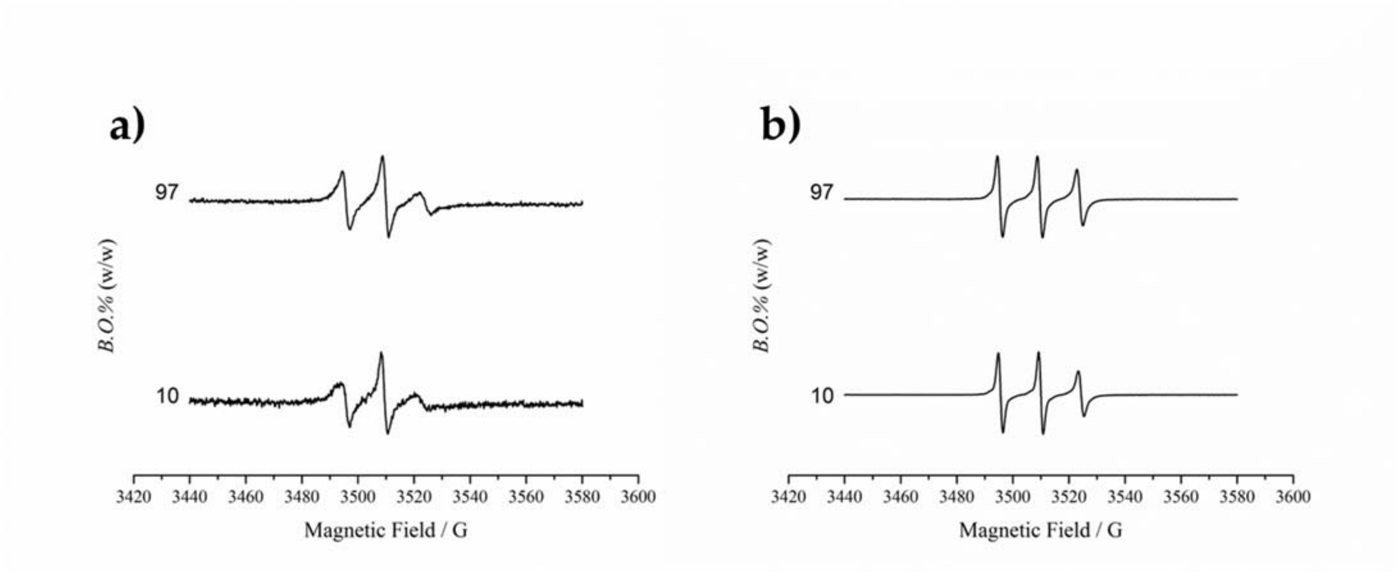

Figure S3. Electron paramagnetic resonance (EPR) spectra of 5-DSA (**a**) and 16-DSA (**b**) in water/B.O./ (Tween80+Span80) emulsions at 25 °C, recorded within 3 h from sample preparation. The total surfactant amount was fixed to 4% in weight with respect to the total sample amount and surfactant compositions was α_{Span80} = 0.4 in all samples. The oil content is indicated in the labels of the figures.
